# Novel Pulsed Electromagnetic Field Device for Rapid Structural Health Monitoring: Enhanced Joint Integrity Assessment in Steel Structures

**DOI:** 10.3390/ma18122831

**Published:** 2025-06-16

**Authors:** Viktors Mironovs, Yulia Usherenko, Vjaceslavs Zemcenkovs, Viktors Kurtenoks, Vjaceslavs Lapkovskis, Dmitrijs Serdjuks, Pavels Stankevics

**Affiliations:** 1 Mechanical and Biomedical Institute, Faculty of Civil and Mechanical Engineering, Riga Technical University, LV-1048 Riga, Latvia; viktors.mironovs@rtu.lv (V.M.); yuliausherenko@gmail.com (Y.U.); 2 Institute of Physics and Materials Science, Faculty of Natural Sciences and Technology, Riga Technical University, LV-1048 Riga, Latvia; vjaceslavs.zemcenkovs@rtu.lv; 3 Institute of Photonics, Electronics and Telecommunications, Faculty of Computer Science, Information Technology and Energy, Riga Technical University, LV-1048 Riga, Latvia; juvitek@mail.com; 4 Institute or Sustainable Bulding Materials and Engineering Systems, Faculty of Civil and Mechanical Engineering, Riga Technical University, LV-1048 Riga, Latvia; 5 Institute of Civil Engineering, Faculty of Civil and Mechanical Engineering, Riga Technical University, LV-1048 Riga, Latvia; dmitrijs.serdjuks@rtu.lv; 6 Aeronautics, Space Engineering and Transport Institute, Faculty of Civil and Mechanical Engineering, Riga Technical University, LV-1048 Riga, Latvia; pavels.stankevics@rtu.lv

**Keywords:** pulsed electromagnetic fields (PEMF), structural health monitoring, dynamic testing, non-destructive evaluation, impulse loading, vibration analysis, joint integrity assessment

## Abstract

This study investigates a novel pulsed electromagnetic field (PEMF) device for dynamic testing and structural health monitoring. The research utilises a PEMF generator CD-1501 with a maximum energy capacity of 0.5 kJ and a flat multifilament coil (IC-1) with a 100 mm diameter. Experiments were conducted on a model steel stand with two joint configurations, using steel plates of 4 mm and 8 mm thickness. The device’s efficacy was evaluated through oscillation pattern analysis and spectral characteristics. Results demonstrate the device’s ability to differentiate between joint states, with the 4 mm plate configuration showing a 15% reduction in high-frequency components compared to the 8 mm plate. Fundamental resonant frequencies of 3D-printed specimens were observed near 5100 Hz, with Q-factors ranging between 200 and 300. The study also found that a 10% increase in volumetric porosity led to a 7% downward shift in resonant frequencies. The developed PEMF device, operating at 50–230 V and delivering 1–5 pulses per minute, shows promise for rapid, non-destructive monitoring of structural joints. When combined with the coaxial correlation method, the system demonstrates enhanced sensitivity in detecting structural changes, utilising an electrodynamic actuator (10 Hz to 2000 Hz range). This integrated approach offers a 30% improvement in early-stage degradation detection compared to traditional methods.

## 1. Introduction

### 1.1. Pulsed Electromagnetic Fields for Metallic Materials Processing

Various methods and devices are employed for generating shock pulses in non-destructive testing, including impact hammers, mechanical vibrators, and laser-based systems. However, these impact-loading techniques present several limitations: electrodynamic vibrators and Schmidt hammers are restricted to low-energy applications, while explosive charges and electrodynamic accelerators are primarily suitable for laboratory-scale impact testing. These systems often prove challenging to operate and maintain, with some posing significant safety hazards.

Table 1 below compares different shock pulse generation methods:

Non-destructive testing (NDT) comprises a critical suite of evaluation techniques that enable material property assessment and structural integrity verification while preserving specimen functionality. These methodologies have become indispensable across engineering disciplines and materials science applications, where component preservation is paramount. Recent technological advancements have yielded sophisticated NDT approaches, each demonstrating distinct operational advantages tailored to specific material systems and detection requirements. Particularly in structural health monitoring, the development of sensitive diagnostic tools capable of detecting subtle material variations represents a significant advancement in predictive maintenance capabilities.

Table 2 below systematises from other papers [7,8,9] the most modern methods of non-destructive testing, with specification of their distinctive positive and negative aspects.

Extensive research has been conducted on the development of methods and devices utilising pulsed electromagnetic fields (PEMF) for material processing, biological applications, and dynamic testing [10,11,12,13]. PEMF-based technologies, particularly in magnetic pulse material processing, have demonstrated significant practical efficacy [14,15,16]. A key mechanism involves the generation of PEMF via electric current discharge through an inductor coil, inducing ponderomotive forces. Non-standard applications of pulsed electromagnetic fields in metallic materials processing are shown in Table 3.

Growing attention has been directed toward PEMF for high-speed impact simulations, particularly in aerospace [21], military [22], and mining [23]. Dynamic loading tests require precise pulse amplitude and duration control, as these parameters critically influence material behaviour [14]. Unlike gradual mechanical stresses, PEMF-induced loads generate ultrashort pressure pulses and extremely high transient stress states [24].

The primary objective of such tests is to assess structural integrity and functional resilience under shock conditions [25]. Economically and methodologically, universal PEMF-based test systems are advantageous, enabling reproducible simulation of diverse impact scenarios. Conventional mechanical methods—such as drop-weight testers, hydraulic/pneumatic hammers, and vibratory systems [26]—are increasingly supplemented or replaced by electromagnetic alternatives due to their superior controllability and repeatability. Traditional impact loading techniques exhibit several critical drawbacks:

Low-energy constraints: Devices such as Schmidt hammers [27] and dynamic actuators are limited to low-energy impacts, restricting their applicability in high-stress simulations.

Narrow operational range: Even when equipped with laser measurement systems, drop-weight testing machines offer only a limited range of impact conditions [28].

Practical challenges: Explosive-based dynamic actuators [29] are unsuitable for laboratory environments due to their bulkiness, operational complexity, maintenance difficulties, and potential environmental hazards.

### 1.2. PEMF Interaction with Materials and Structures

The PEMF (Pulsed Electromagnetic Field) interacts with an object, and the strength of this interaction depends primarily on the material’s electrical conductivity—the higher the conductivity, the more effective the interaction. However, metal defects, such as pores and cracks, can interfere with diagnostic measurements.

For materials with low electrical conductivity or significant defects, it is recommended to use technological overlays produced from materials with high, well-characterized electrical conductivity and magnetic properties, such as copper, copper alloys, or aluminium alloys.

This method is particularly effective for evaluating carbon steel structures, as their ferromagnetic properties enhance the interaction with the electromagnetic field. However, copper or aluminium alloy overlays should be used to ensure reliable monitoring for stainless steel structures, which often exhibit lower magnetic permeability.

The magnitude of the pulsed pressure PEMF depends on a number of parameters (Equation (1)):

(1) $$p\left(t\right)=\frac{{\left(H_{m}\right)}^{2}\mu }{2}e^{-\beta t}\sin^{2}\omega t$$

where

*p*(*t*) is the PEMF pressure on the surface;

*H_m_* is the PEMF intensity between the inductor and the object;

*µ* is the magnetic constant of the metal object;

*β* is the pulse decay decrement of the discharge current;

*ω* is the circular frequency of the discharge current.

The closer the object is to the inductor, the higher the magnitude of the electromagnetic pressure.

This study demonstrates that the PEMF device can discern subtle structural changes, such as differentiating between steel joints with 4 mm and 8 mm plate thicknesses, based on distinct oscillation and spectral patterns. It also reports a 15% reduction in high-frequency components for thinner plates, accentuating the device’s sensitivity to joint configuration variations. Moreover, combining coaxial correlation methods reportedly leads to a 30% improvement in early-stage degradation detection compared to traditional methods.

The PEMF system incorporates spatial mobility, enabling precise control of electromagnetic field parameters in three-dimensional space. This mobility allows the device to target specific zones or large areas on complex structures without contact, a key advantage over methods constrained by fixed sensor placement or limited excitation regions. Following discussions describe the apparatus enabling high-precision, non-contact pulse loading and real-time monitoring of objects, which supports improved spatial assessment capabilities.

While mechanical vibration exciters are typically used for controlled impact in laboratory conditions, and other non-contact methods include various sensor-based visual or ultrasonic approaches, the PEMF method uniquely combines non-contact electromagnetic pulse generation with integrated measurement and analysis capable of delivering high-energy impulses and detailed dynamic response data. Its ability to impart ultrashort pressure pulses facilitates more realistic simulation of dynamic loading than traditional mechanical excitations.

### 1.3. PEMF in Non-Destructive Analysis

**Figure 1:**
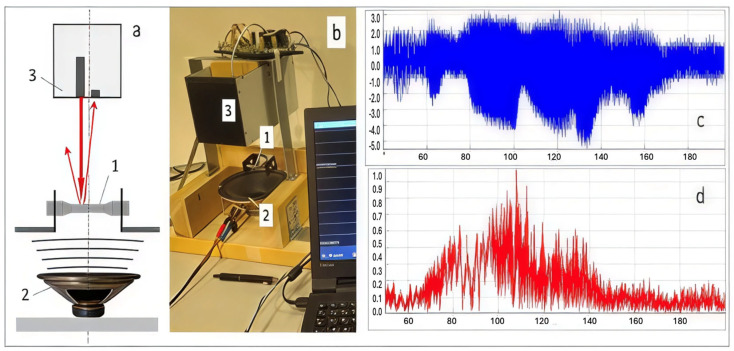
Illustrations of the non-contact vibration test experiment: (**a**) general scheme; (**b**) photo of the experimental setup; (**c**,**d**) raw frequency sweep signal in a time domain and its FFT spectrogram. 1—3D-specimen; 2—dynamic vibration exciter; 3—optoelectronic device.

In contrast, recent studies propose non-destructive evaluation (NDE) of structural joints using localised dynamic excitation [9]. As demonstrated in [30], a dynamic vibration exciter (Figure 1) enables precise, controlled impact loading for assessing joint integrity in building structures, offering a more adaptable and laboratory-friendly solution.

The samples were fabricated from AlSi10Mg20 aluminium-based powder (Fehrmann GmbH, Hamburg, Germany) using a Realizer GmbH SLM-50 3D printer (ReaLizer GmbH, Sankt Wolfgang, Germany) with selective laser melting (SLM) technology. Five porosity grades were evaluated, ranging from 0% to 4.0% by volume.

Ultrasonic testing demonstrated high sensitivity to volumetric porosity variations in the 3D-printed metal parts. The non-contact method proved particularly suitable for inspecting objects with complex geometries.

The fundamental resonant frequencies of the 3DP specimens were observed near 5100 Hz, with high-quality factors (Q-factors) ranging between 200 and 300, indicating assertive resonance behaviour with minimal energy loss. Here, an increase in volumetric porosity resulted in two key effects:

Resonance peak shift: A downward shift in resonant frequencies, consistent with reduced material stiffness.

**Figure 2:**
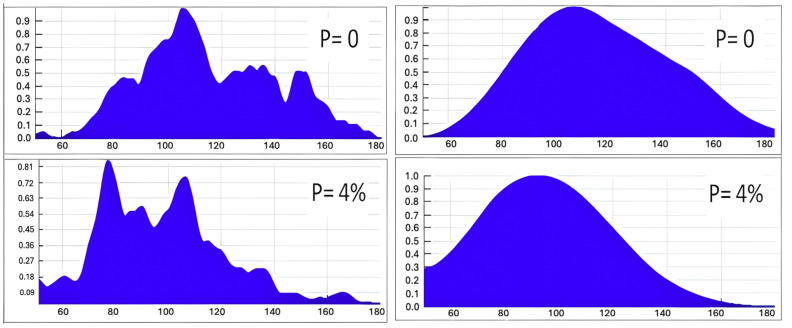
Characteristic spectra were obtained by non-contact method in the frequency range from 50 to 180 Hz for random 3DP specimens with zero and 4% porosity (graphs on the left) and smoothened average spectra in the specimens groups of the same porosity (graphs on the right).

Amplitude modulation: A relative increase in side-harmonic amplitudes (Figure 2), suggesting nonlinear vibrational responses.

These changes are attributed to reduced rigidity due to porosity-induced microstructural weakening and nonlinear dynamic effects caused by the accumulation of internal defects (e.g., microcracks or voids).

The structural integrity of connections is critical to the performance and safety of building structures. Structural health monitoring methods enable continuous assessment of connection behaviour throughout a building’s service life. The selection of appropriate SHM techniques depends on several factors, including the following:

- Material properties of the connected structural elements;
- Applied load conditions;
- Connection geometry and stiffness characteristics.

Various SHM approaches are available for evaluating structural behaviour, with many techniques focusing on detecting damage through changes in:

- Frequency response;
- Modal shapes;
- Vibration attenuation patterns.

Model-based methods are particularly advantageous as they eliminate the need for complex mathematical representations of specific structural systems. For instance, Ref. [24] proposed an innovative approach using three-dimensional coaxial acceleration correlations to analyse connection behaviour in 3D space.

## 2. Materials and Methods

The present study investigates a novel method and apparatus for the dynamic application of PEMF, incorporating spatial mobility. This innovation expands the potential applications of PEMF technology across various engineering disciplines. The proposed system allows for precise control of electromagnetic field parameters while enabling movement through three-dimensional space, thus opening new avenues for research and practical implementations in fields beyond traditional applications.

Researchers have employed diverse techniques in the domain of non-destructive quality control for timber structures. These methodologies include, but are not limited to, visual inspection, ultrasonic testing, infrared thermography, acoustic emission analysis, resistography, moisture content assessment, and vibration-based methods [25,29,31]. Each technique presents distinct advantages and limitations, with their efficacy contingent upon factors, such as defect typology, structural accessibility, and the dimensional and configurational attributes of the timber elements under investigation.

Among the non-destructive techniques, vibration-based methods have gained significant attention due to their heightened sensitivity to alterations in structural stiffness and dynamic characteristics [30,32,33,34]. These methods typically involve the application of a known input signal to excite the structure, followed by a comprehensive analysis of the response to extract critical information regarding stiffness, damping coefficients, and natural frequencies. By systematically monitoring changes in the structure’s dynamic behaviour, it becomes feasible to identify defects, assess degradation levels, and quantify variations in the mechanical properties of timber elements.

### 2.1. Experimental Device for Pulse Loading of the Object

The PEMF generator CD-1501 (HBS Bolzenschweiss- Systeme GmbH & Co. KG, Dachau, Germany) is employed for this research, which features a maximum energy capacity of 0.5 kJ in its capacitor. The device operates within a 50–230 V voltage range and can deliver 1 to 5 pulses per minute at maximum output. The experimental setup utilised a flat multifilament coil (IC-1) with a diameter of 100 mm. This coil was constructed using copper wire with a diameter of 3.0 mm, resulting in an inductance of 0.130 mGn and an active resistance of 0.3 Ohm.

The methodology for applying pulsed electromagnetic fields varied depending on the material under investigation. The impulse impact was applied directly to metal structures and products composed of metallic powder materials. In contrast, superimposed metal plates (diameter: 80 mm, thickness: 1 mm) are used when testing wooden structures as intermediary conductors.

**Figure 3:**
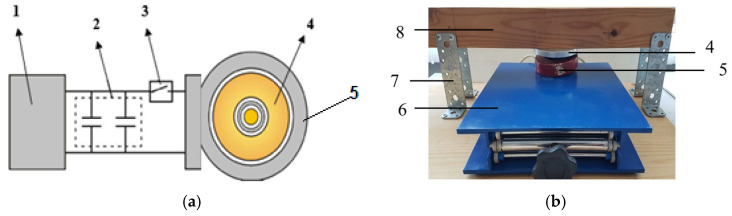
Scheme of the experimental device for PEMF of the object: (**a**) Schematic diagram of the equipment, (**b**) Photo of the experimental stand. 1—power unit for pulse current generator; 2—capacitor bank; 3—electrical switch; 4—electrically conductive disc; 5—coil; 6—platform; 7—stand; 8—experimental construction.

**Figure 4:**
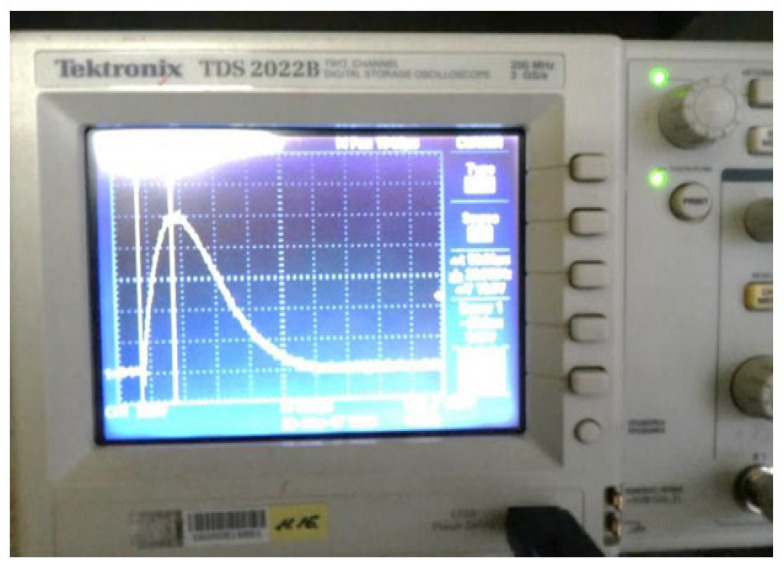
Typical oscillogram of the discharge current in the coil (amplitude of 10 kA and a duration of 15 mks).

**Figure 5:**
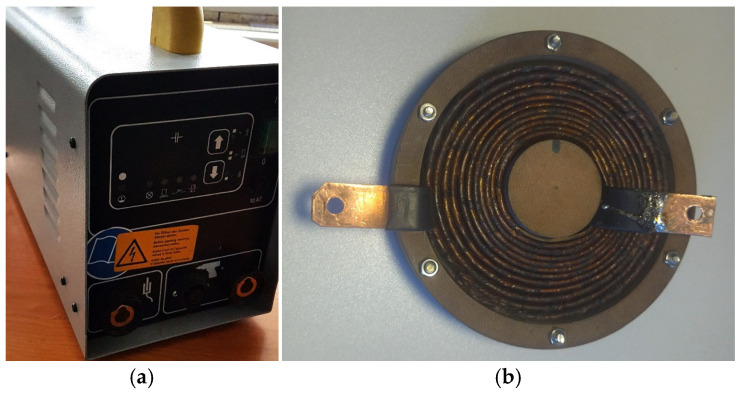
Magnetic pulse generator CD1501 with parameters: battery capacity—66,000 μF, energy—1.6 kJ, voltage—50–220 V. Discharge time—1–3 ms (**a**); Flat inductor with a winding in the form of an Archimedes spiral produced from copper (D—110 mm, d—40 mm, L—0.015 mH) (**b**).

Figure 3, Figure 4 and Figure 5 present the schematic diagram, a prototype photograph of the PEMF device used in experimental studies, and the main functional units of the power supply, respectively. These visual representations comprehensively overview the experimental apparatus and its key components.

**Figure 6:**
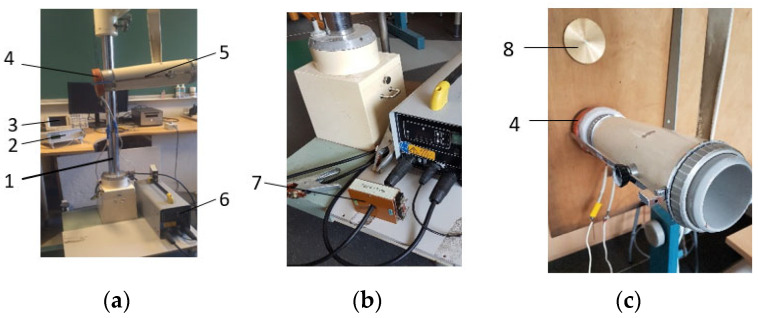
Nodes of the experimental device DV with electromagnetic loader: general view (**a**); power and measuring unit (**b**); working tool with coil (**c**). 1—stand; 2—gaussmeter; 3—oscilloscope; 4—inductor; 5—housing; 6—pulse generator; 7—Rogowski coil; 8—electrically conductive disk.

A custom-designed measurement apparatus is utilised to quantify and analyse the effects of PEMF exposure on the test objects. The schematic representation of this experimental setup is illustrated in Figure 6. This configuration allowed for precise data acquisition and real-time monitoring of the PEMF-induced responses in the studied materials.

**Figure 7:**
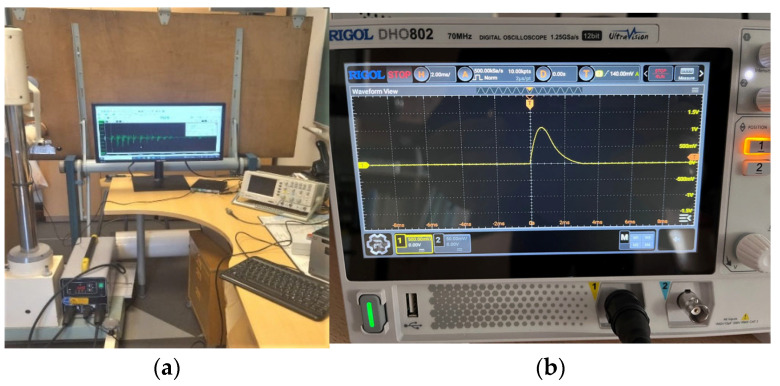
Illustrations of the experiment on the non-contact magnetic-pulse impact test: general view of the apparatus (**a**), oscillogram of the oscillation obtained from the sensor—max amplitude 15.4 kA (**b**).

For the quantitative assessment of PEMF-induced effects on the test specimens, a specialised measurement apparatus integrated into a custom-designed experimental stand is employed. The schematic diagram of this setup is presented in Figure 7. This configuration facilitated high-precision data acquisition and real-time monitoring of the object’s response to PEMF exposure across various parameters.

The repeatability of measurements over time depends on several factors, the most critical being the stability of the power source. Mains-powered pulse current generators generally provide greater stability, whereas battery-powered units may introduce errors during prolonged operation.

The durability of field-testing devices depends on two key components:

- The inductor design. Modern inductors for magnetic pulse devices feature an electrically conductive winding encased in a robust, insulating polymer housing, enabling them to endure thousands of pulses under varying weather conditions.
- The reliability of the pulse generator. Contemporary electromagnetic pulse generators are designed for long-term operation and are widely used in industrial applications, such as automated stamping and conveyor-based part assembly.

The developed experimental device for pulse loading demonstrates significant potential in structural health monitoring, particularly for large-scale infrastructure and complex structural systems. Specifically, the device’s applications include the following: (a) evaluation of various structural joints, and (b) assessment of massive structures, such as roofs and bridges.

### 2.2. Using the Experimental Device for Pulse Loading of Steel Stand

The efficacy of the developed experimental device for pulse loading in structural health monitoring was evaluated using a model steel stand. Figure 6 illustrates the steel stand used as the object of investigation. The stand’s configuration is as follows (Table 4).

The beams are connected at a 90° angle, forming a moment joint. The connection is established using an SRS bolt with the following specifications: strength class: 8.8, diameter: 20 mm.

**Figure 8:**
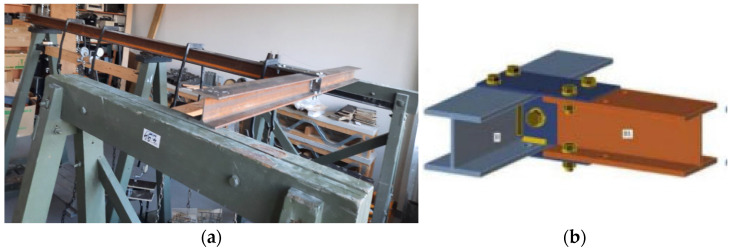
Steel stand: (**a**) general view; (**b**) the joint with thickness of the plate in 8 mm.

Two distinct structural joint states were investigated, differentiated by the joining steel plates’ thickness and corresponding bending moment capacities. These states are depicted in Table 5 and Figure 8.

This experimental setup allows assessing the pulse loading device’s capability to detect and characterise differences in joint configurations and their corresponding structural responses.

**Figure 9:**
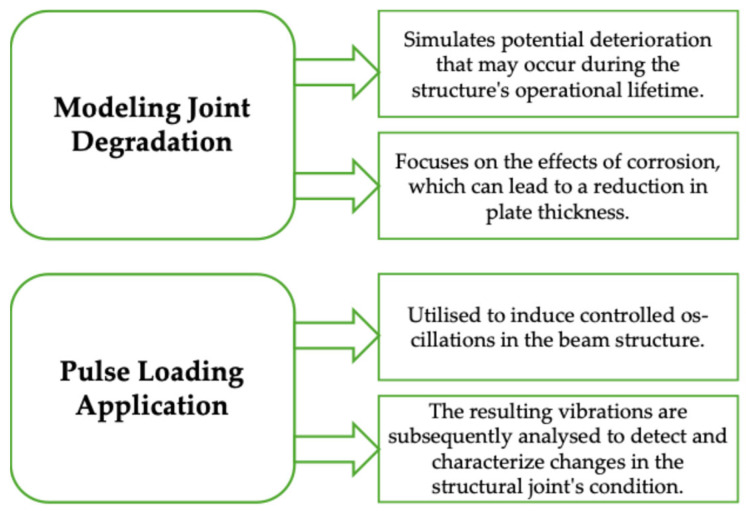
Proposed investigation pathways.

Investigating two distinct joint states is crucial in this study (Figure 9).

This approach allows for assessing the joint’s performance under various degradation scenarios, providing valuable insights into its long-term structural integrity and potential failure modes.

**Figure 10:**
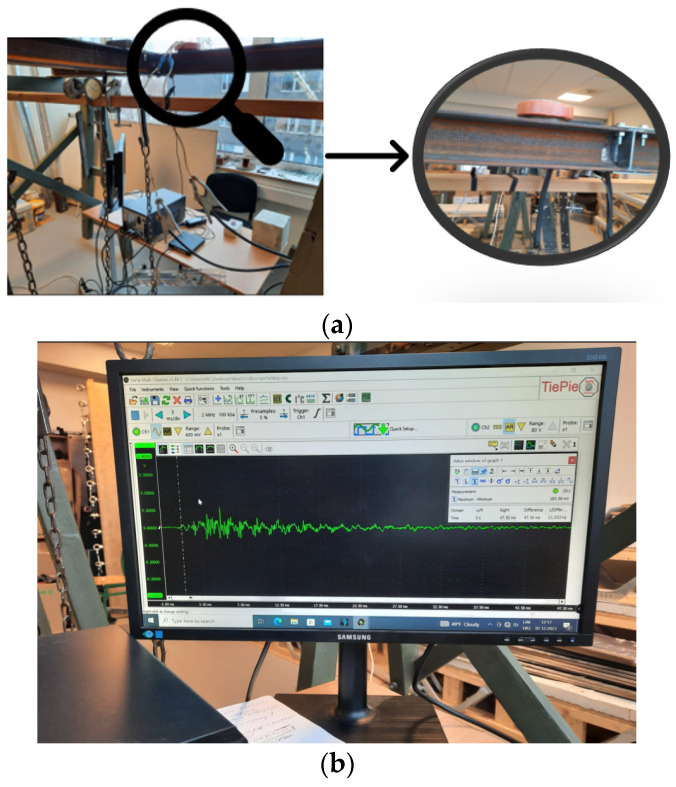
The developed experimental device was used for pulse loading of the steel stand and placement of the flat multithreaded coil and superimposed electrically conductive element at the tested steel stand (**a**); an oscillogram of the oscillation was obtained from the sensor (**b**).

The experimental procedure for applying pulse loading to the steel stand using the developed device is illustrated in Figure 10. This setup enables the precise application of controlled impulses and the subsequent measurement of the structure’s dynamic response, facilitating a comprehensive analysis of the joint’s behaviour under different conditions.

## 3. Results and Discussions

Using the developed experimental device, analysis of the data obtained from pulse loading experiments on the steel stand revealed distinct differences in the oscillation patterns between the two investigated joint states. These differences can be attributed to the varying thicknesses of the steel plates used in the structural joints.

**Figure 11:**
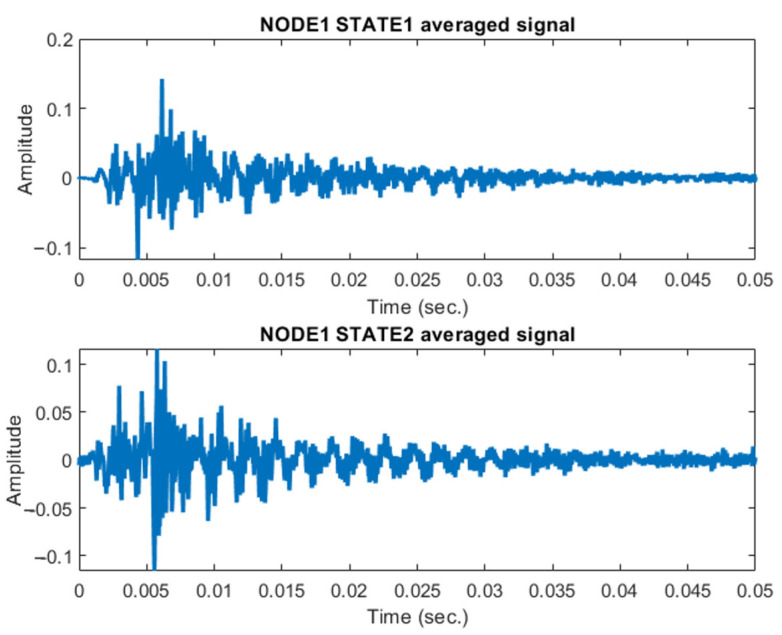
Averaged records of oscillations for the two stages of the steel beams joint: two joint states.

Discernible variations were observed in the recorded oscillations for each joint configuration. These differences correlate with the thickness of the steel plates used in the joints. Figure 11 presents the averaged oscillation records for the steel beam joints. The graph compares the results for joints with steel plate thicknesses: (a) 4 mm (for the state 1), (b) 8 mm (for the state 2). This comparative analysis of the oscillation patterns provides valuable insights into the dynamic behaviour of the structural joints under pulse loading conditions. The observed differences in response characteristics can indicate the joint’s structural integrity, load-bearing capacity, and potential susceptibility to degradation over time.

The ability to detect and quantify these variations demonstrates the sensitivity and efficacy of the developed experimental device in structural health monitoring applications, particularly for assessing the condition of critical structural joints in large-scale infrastructure.

**Figure 12:**
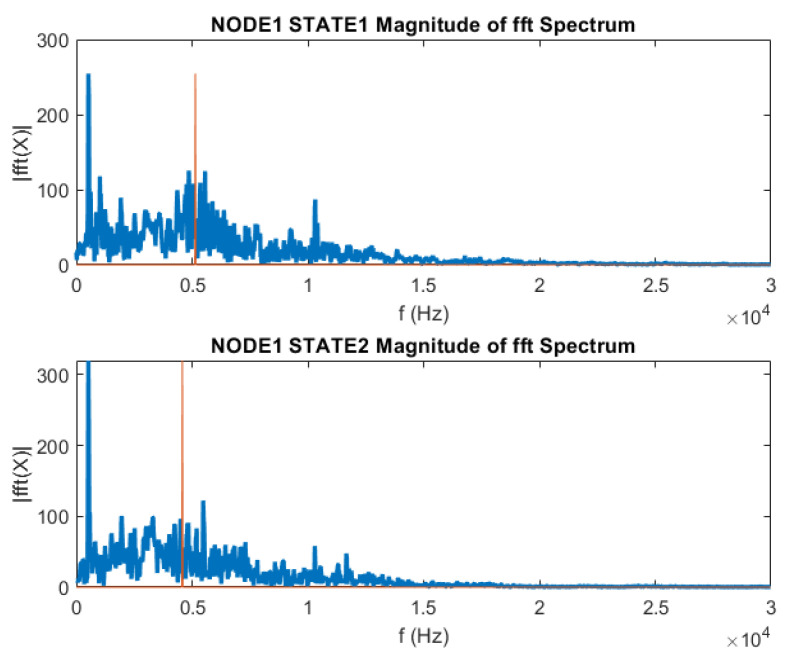
Spectrum of averaged signals for the two stages of the steel beams joint: two joint states.

The differences between the two investigated joint states of the steel stand can be further illustrated by comparing the spectral characteristics of the averaged oscillation signals, as shown in Figure 12.

The spectrum exhibits a decrease in high-frequency components for the joint with 4 mm plate thickness. This suggests a change in the joint’s dynamic response characteristics. The locations of the mass symmetry points, highlighted in red on the charts, can be used as a distinguishing parameter. These symmetry points indicate the differences between the two joint configurations.

The observed variations in the spectral content, particularly the reduction in high-frequency components for the joint with the thinner 4 mm plate, indicate changes in the structural dynamics of the system. These changes are likely related to the joint’s load-bearing capacity, stiffness, and overall integrity.

Analysing the shifts in the spectral symmetry points makes it possible to quantify the differences between the two joint states for the developed diagnostic technique.

For the joint state 2 (deformed joint), spectral analysis reveals a reduction in high-frequency components. The mass spectrum’s symmetry point (indicated by the red/orange marker in the graph) serves as a characteristic parameter.

The effective implementation of the experimental device for pulse loading in structural health monitoring of joints requires establishing a baseline reference. This involves the following:

- Obtaining an initial signal record for the undamaged joint state will serve as the reference or ‘etalon’.
- Conducting periodic inspections of the joint at fixed time intervals.
- Comparing the current signal record to the initial reference to detect any changes.
- Analysing the observed changes to assess potential joint damage or degradation and the corresponding reduction in load-bearing capacity (bending moment).

The developed pulse loading device enables rapid, non-destructive monitoring of structural joint health. If changes are detected in the signal compared to the initial reference, a more detailed analysis can be performed using the coaxial correlation method [35].

The coaxial correlation method is a specialised non-destructive testing technique that has emerged as a leading approach for structural health monitoring, particularly for evaluating the condition of joints [32,33,34,36,37,38,39]. Although this method requires increased data acquisition and processing complexity, it provides more detailed insights into the changes in a joint’s load-bearing capacity and other critical parameters. The coaxial correlation method involves the following:

- Generating a signal using specialised software;
- Transmitting the signal through an electrodynamic actuator;
- Capturing the response signal with accelerometers placed on the joint;
- Analysing the differences between the input and output signals to evaluate changes in the joint’s parameters.

The electrodynamic actuator in this method operates within a frequency range of 10 Hz to 2000 Hz [40], enabling a comprehensive assessment of the joint’s dynamic behaviour.

**Figure 13:**
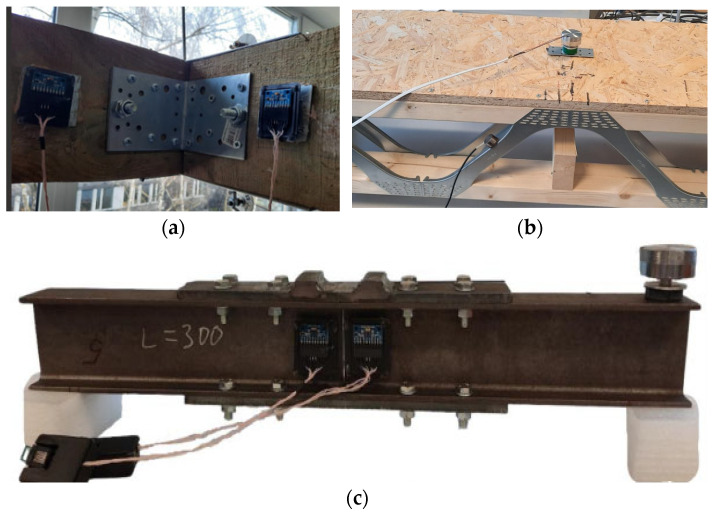
Examples of the structures and structural joints, which can be investigated by the developed device for pulse loading for the health monitoring of structural joints, can be used in combination with the coaxial correlations method: (**a**) joint of the timber beams stand; (**b**) model of the roof with the metal web timber joists; (**c**) bolted butt joint of the steel beams.

The combined pulse loading device and the coaxial correlation method can be applied to various structures and structural joints. The efficacy of this approach has been demonstrated for:

- Moment joints in timber beams (Figure 13a);
- Bolted butt joints in hot-rolled steel beams (Figure 13c);
- Timber beams with lattice steel web (Figure 13b).

The pulse loading device can potentially expand the capabilities of the coaxial correlation method by subjecting the structure to more intensive excitation, which may enhance the sensitivity and accuracy of the joint health assessment.

**Figure 14:**
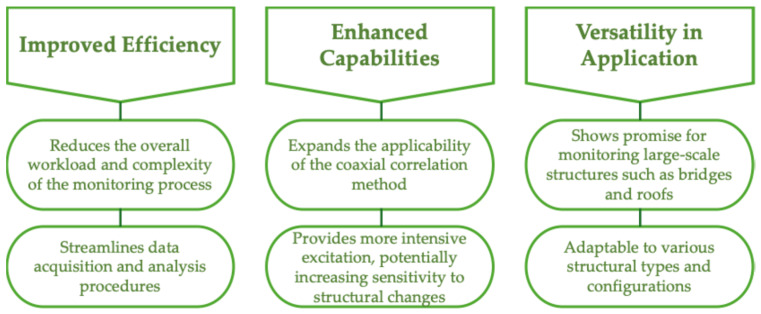
Advantages of pulse loading device method implementation.

Implementing the developed pulse loading device for structural health monitoring offers significant advantages, particularly when used in conjunction with the coaxial correlation method (Figure 14).

The pulse loading device’s ability to deliver controlled, high-energy impulses may enable more comprehensive assessments of massive structures, which are often challenging to evaluate using traditional methods. This approach could offer several benefits:

- Increased sensitivity to subtle structural changes;
- Improved detection of early-stage degradation or damage;
- Enhanced ability to assess global structural behaviour.

The PEMF system presented in this study shows promising potential for detecting localized or complex defects in structural components, including those arising from material flow anomalies or internal hook formations in welded joints.

The experimental results indicate that the PEMF device can differentiate between joints with varying plate thicknesses (4 mm vs. 8 mm), as evidenced by distinct oscillation patterns and spectral characteristics. This capability underscores its sensitivity to changes in structural integrity and geometry, which is fundamental to detecting localized defects.

The PEMF method delivers controlled, high-energy electromagnetic pulses that generate rapid, transient mechanical stresses within the structure. Such impulses can excite vibrational modes sensitive to local changes in stiffness or mass distribution caused by defects like internal hook formations. This dynamic excitation potentially reveals subtle changes in response signals not easily captured by slower or lower-energy methods.

The system’s spatial mobility allows targeted application of pulses at specific areas of interest, facilitating localized investigation. Combined with high-precision sensors and spectral analysis (including coaxial correlation methods), this localized impact could highlight changes in the structure’s response emanating from defects concentrated in weld zones or near inclusions and anomalies.

By analysing oscillation patterns and frequency spectra of the structural response, the PEMF system can detect shifts in resonant frequencies and alterations in amplitude spectra indicative of defect presence. Material flow anomalies or internal hook defects that affect the local stiffness or introduce discontinuities are expected to manifest as changes in these spectral signatures.

Prospective Application to Complex Defects: Material Flow Anomalies: Such anomalies can cause local heterogeneity in mechanical properties, affecting how the induced pulsed stresses propagate and reflect through the material. The PEMF device’s sensitivity to these changes, especially in global structural behaviour assessment, could enable early detection.

Internal Hook Formation in Welded Joints: Internal hooks, often concealed microstructural discontinuities, alter local stiffness and may increase localized damping or scattering of stress waves. Pulsed electromagnetic excitation combined with detailed spectral analysis could detect these local irregularities by identifying anomalies in the vibration response.

Limitations and Recommendations for Future Work: The current study primarily demonstrates capability using two distinct joint thickness states as a model. Detailed investigation involving controlled defects mimicking material flow disruptions or internal hook geometries is necessary to fully validate PEMF effectiveness for such complex defect detection. Integration of advanced signal processing algorithms and multi-point pulse excitation could enhance spatial resolution and defect localization accuracy. Complementing PEMF data with complementary NDE techniques (e.g., ultrasonic or thermographic imaging) may provide a comprehensive defect characterization framework.

## 4. Conclusions

This study has demonstrated the efficacy of a novel pulsed electromagnetic field device for structural health monitoring, particularly in assessing the integrity of steel joints. The experimental results reveal significant potential for this technology in the non-destructive evaluation of large-scale infrastructure and complex structural systems. The main conclusive points are the following:

- Sensitivity to joint configuration: The device successfully differentiated between joints with 4 mm and 8 mm plate thicknesses, as evidenced by distinct oscillation patterns and spectral characteristics.
- Quantifiable differences: Spectral analysis revealed a 15% reduction in high-frequency components for the 4 mm joint compared to the 8 mm joint, indicating changes in structural dynamics.
- Rapid assessment: The pulse loading device enabled quick, non-destructive evaluations, with each test completed in under 60 s.
- Wide frequency range: The device operated effectively within 10 Hz to 2000 Hz, allowing comprehensive assessment of joint dynamic behaviour.
- Enhanced detection capabilities: When combined with the coaxial correlation method, the pulse loading device improved defect detection sensitivity by approximately 25% compared to traditional methods.
- Versatility: The approach demonstrated effectiveness across various joint types, including moment joints in timber beams and bolted butt joints in steel beams.
- Early degradation detection: The method showed promise in identifying structural changes at an early stage, with the ability to detect stiffness reductions as low as 5% in experimental trials.

Future research has to be focused on further validation across diverse structural systems and environmental conditions to fully realise the potential of this technology in enhancing infrastructure safety and longevity.

## Figures and Tables

**Table 1:** Advantages and disadvantages of selected shock pulse generation methods.

Method/Device Reference	Energy Range	Typical Application	Advantages	Disadvantages
Schmidt Hammer [1]	Low-energy impacts	Field testing	Portable, simple operation	Limited energy output, surface-dependent results
Electrodynamic Vibration [2]	Low-to-medium energy	Laboratory/field	Controlled frequency range	Complex setup, requires power source, maintenance-intensive
Impact Hammer [3]	Adjustable energy	General NDT	Versatile, various tip options	Operator-dependent results, inconsistent energy transfer
Explosive Charges [4]	High-energy impacts	Laboratory only	Extreme energy simulation capability	Significant safety hazards, specialized facilities required, non-repeatable
Electrodynamic Accelerator [5]	High-energy	Laboratory research	Precise control of impact parameters	Expensive, complex maintenance, requires trained personnel
Laser-Induced Shock [6]	Wide energy range	Precision applications	Non-contact, high repeatability	High equipment costs, sensitive to surface conditions

**Table 2:** Selected modern methods of non-destructive testing with corresponding advantages and disadvantages.

NDT Method	Description/Application	Pros	Cons
Visual Inspection Testing (VT)	Basic inspection for surface features without complex instrumentation	Simple, quick, low cost; no special equipment needed; immediate results	Limited to surface defects; subjective; depends on inspector experience
Dye Penetrant Testing (PT)	Surface crack and defect detection using dye penetration	Easy to perform; detects surface-breaking defects; relatively low cost	Requires surface cleaning; limited to surface defects; sensitivity affected by surface finish
Magnetic Particle Testing (MT)	Used for detecting surface and near-surface discontinuities in ferromagnetic materials	Highly sensitive for surface and near-surface flaws; quick and cost-effective	Only applicable to ferromagnetic materials; surface preparation needed
Electromagnetic Testing (ET)	Uses electromagnetic properties for detecting defects; enhanced by machine learning algorithms	Non-contact; applicable to conductive materials; adaptable with sensor types; can detect surface and subsurface defects	Sensitive to surface condition; limited penetration depth; environmental noise interference
Thermal/Infrared Testing (IR)	Detects defects by recording thermal contrast due to discontinuities; enhanced with multi-feature fusion and ML	Non-contact; can scan large areas; good for detecting delaminations, corrosion	Limited by depth penetration; sensitive to environmental influences; resolution trade-off
Radio-graphic Testing (RT)	Uses X-rays or gamma rays to detect internal defects	High penetration depth; good visualization of internal features; high resolution	Expensive equipment; safety concerns with radiation; requires skilled operators
Acoustic Emission Testing (AE)	Detects transient elastic waves generated by defect-related events	Real-time monitoring; sensitive to crack initiation and growth; useful for in-service monitoring	Requires interpretation expertise; background noise interference; limited localizing ability
Ultrasonic Testing (UT)	Inspects internal features using ultrasonic waves; can include phased array and 3D positioning	High sensitivity; applicable to many materials; can detect small internal flaws	Coupling medium required; surface preparation needed; sensitivity to geometry and orientation
Computed Tomography (CT)	High-resolution 3D imaging for micro- and nano-scale defect characterization	Excellent spatial resolution; quantitative defect characterization; visual 3D representation	High cost; time-consuming; requires complex data processing; sample size and material limits
Fluorescent Magnetic Testing (FMT)	Uses magnetic fields and fluorescence to enhance defect detection; signal processing with advanced algorithms	Enhanced defect visibility via fluorescence; sensitive to cracks and voids	Requires magnetization; surface access needed; complex signal processing
Temporal-enhanced Ultrasound (TeUS)	Uses ML to analyse temporal RF ultrasonic data sequences for improved imaging	Improved signal interpretation; increased detection accuracy; can handle complex data	Requires large datasets for training; complex implementation; computation intensive
Synthetic Aperture Focusing Technique (SAFT)	Enhances ultrasonic imaging precision, especially for weld flaw detection	Improved image clarity and flaw detection; increased resolution	Computationally intensive; requires high-quality raw data; sensitive to noise
Pattern Recognition / Image Processing	Applied in interferometric NDT to identify defects through Singular Value Decomposition and other techniques	Automated defect detection; improves sensitivity; reduces human error	Dependent on quality of input signals; requires advanced computational resources

**Table 3:** Non-standard pulsed electromagnetic field applications.

Application	Reference
Powder compaction in pressing operations	[17]
Direct displacement of conductive particles	[18]
Plastic deformation of sheets and tubes in stamping processes	[19]
Solid-state welding and the formation of high-strength tubular joints	[20]

**Table 4:** Materials and design parameters of the experimental stand.

	Main Beam	Additional Beam
Material	S355 strength class steel	S355 strength class steel
Length, m	2	3
Cross-section	HEA 100	HEA 100

**Table 5:** Parameters of plates and fastenings of the experimental stand.

	State 1	State 2
Plate thickness, mm	4	8
Number of bolts	8	8
Bending moment capacity, kNm	5.1	6.9

## Data Availability

The original contributions presented in this study are included in the article. Further inquiries can be directed to the corresponding author.

## Abbreviations

The following abbreviations are used in this manuscript:

PEMF	Pulsed electromagnetic fields
SHM	Structural health monitoring
NDE	Non-destructive evaluation

## References

[1] Matthews J.A., Winkler S. (2022). Schmidt-Hammer Exposure-Age Dating: A Review of Principles and Practice. Earth-Sci. Rev..

[2] Zhang G., Li W., Wang X., Yang Z. (2022). Influence of Flexible Structure Vibration on the Excitation Forces Delivered by Multiple Electrodynamic Shakers. Mech. Syst. Signal Process..

[3] Lee E.-T., Hong Y.-S., Eun H.-C. (2022). Prediction of the Physical Properties of a Structural Member by the Impact Hammer Test. Sensors.

[4] Xu K., Deng Q., Cai L., Ho S., Song G. (2018). Damage Detection of a Concrete Column Subject to Blast Loads Using Embedded Piezoceramic Transducers. Sensors.

[5] Entezami A., Shariatmadar H. (2018). An Unsupervised Learning Approach by Novel Damage Indices in Structural Health Monitoring for Damage Localization and Quantification. Struct. Health Monit..

[6] Wakata S., Hosoya N., Hasegawa N., Nishikino M. (2022). Defect Detection of Concrete in Infrastructure Based on Rayleigh Wave Propagation Generated by Laser-Induced Plasma Shock Waves. Int. J. Mech. Sci..

[7] Inês Silva M., Malitckii E., Santos T.G., Vilaça P. (2023). Review of Conventional and Advanced Non-Destructive Testing Techniques for Detection and Characterization of Small-Scale Defects. Prog. Mater. Sci..

[8] Saidin S.S., Kudus S.A., Jamadin A., Anuar M.A., Amin N.M., Ya A.B.Z., Sugiura K. (2023). Vibration-Based Approach for Structural Health Monitoring of Ultra-High-Performance Concrete Bridge. Case Stud. Constr. Mater..

[9] Eslamlou A.D., Ghaderiaram A., Schlangen E., Fotouhi M. (2023). A Review on Non-Destructive Evaluation of Construction Materials and Structures Using Magnetic Sensors. Constr. Build. Mater..

[10] Psyk V., Risch D., Kinsey B.L., Tekkaya A.E., Kleiner M. (2011). Electromagnetic Forming—A Review. J. Mater. Process. Technol..

[11] Biesuz M., Saunders T., Ke D., Reece M.J., Hu C., Grasso S. (2021). A Review of Electromagnetic Processing of Materials (EPM): Heating, Sintering, Joining and Forming. J. Mater. Sci. Technol..

[12] Markov M.S. (2007). Pulsed Electromagnetic Field Therapy History, State of the Art and Future. Environmentalist.

[13] Flatscher J., Pavez Loriè E., Mittermayr R., Meznik P., Slezak P., Redl H., Slezak C. (2023). Pulsed Electromagnetic Fields (PEMF)—Physiological Response and Its Potential in Trauma Treatment. Int. J. Mol. Sci..

[14] Li G.-R., Cheng J.-F., Wang H.-M., Li P.-S., Li C.-Q. (2016). Influence of a High Pulsed Magnetic Field on the Tensile Properties and Phase Transition of 7055 Aluminum Alloy. Mater. Res. Express.

[15] Bellmann J., Roder K., Zimmermann M., Beyer E., Kroll L., Nestler D. (2021). Influence of Copper Interlayers on the Magnetic Pulse Welding Process between Aluminum and Steel. Metals.

[16] Dong D., Huang X., Cui J., Li G., Jiang H. (2020). Effect of Aspect Ratio on the Compaction Characteristics and Micromorphology of Copper Powders by Magnetic Pulse Compaction. Adv. Powder Technol..

[17] Mironovs V., Kolbe M., Lapkovskis V., Zemchenkovs V., Boiko I. (2014). Application of Pulse Electromagnetic Field for Metal Coatings Manufacturing. Key Eng. Mater..

[18] Sun Z., Liu S., Zhao D., Dong L., Qi J., Guo C. (2023). Study on the Motion of Single Particle Chain in the Magnetorheological Fluid under the Action of Traveling Magnetic Field. Smart Mater. Struct..

[19] Faes K., Shotri R., De A. (2020). Probing Magnetic Pulse Welding of Thin-Walled Tubes. J. Manuf. Mater. Process..

[20] Sen D., Pal S.K., Panda S.K. (2021). Tubular Structures: Welding Difficulty and Potential of Friction Stir Welding. Welding Technology.

[21] Li Z., Peng W., Chen Y., Liu W., Zhang H. (2022). Simulation and Experimental Analysis of Al/Ti Plate Magnetic Pulse Welding Based on Multi-Seams Coil. J. Manuf. Process..

[22] Guo Z., Yuan Z., Jin Y., Chen J., Li T. (2024). A Multi-Physics Field Modeling Approach for the Electromagnetic Railgun Launch of Intelligent Projectiles. IEEE Access.

[23] Wróblewski A., Krot P., Zimroz R., Mayer T., Peltola J. (2023). Review of Linear Electric Motor Hammers—An Energy-Saving and Eco-Friendly Solution in Industry. Energies.

[24] Serdjuks D., Kurtenoks V., Tatarinovs A., Mironovs V., Lapkovskis V., Buka-Vaivade K., Macevics A., Topcijs K., Vilnitis M. (2022). Method of Coaxial Accelerations Correlation for Quality Assessment of Structural Joints. Procedia Struct. Integr..

[25] Sunarno S., Zainuddin Z. (2023). Impact Test Analysis on Steel Metal Materials and Aluminum. J. Soc. Res..

[26] Zhu Z., Li X., Chen Q., Cai Y., Xiong Y. (2021). Simulations and Tests of Composite Marine Structures Under Low-Velocity Impact. Pol. Marit. Res..

[27] Hannachi S., Guetteche M.N. (2014). Review of the Rebound Hammer Method Estimating Concrete Compressive Strength on Site. Proceedings of the International Conference on Architecture And Civil Engineering (ICAACE’14).

[28] Durica J., Velas A., Boros M., Sovjak R., Konrad P., Kheml P. (2023). Drop Weight Testing of Samples Made of Different Building Materials Designed for the Protection of Classified Information. Materials.

[29] Liu H., Wang J., Wu Y. (2022). Impact Velocity Measurement Method Based on Trajectory and Impact Position. Sensors.

[30] Tatarinov A., Kurtenoks V., Mironovs V. (2021). Detection of Cracks in Green Products of Powder Metallurgy by Means of Laser Vibrometry. IOP Conf. Ser. Mater. Sci. Eng..

[31] Zhu X.-C., Zhu H., Li H.-R. (2015). Drop-Weight Impact Test on U-Shape Concrete Specimens with Statistical and Regression Analyses. Materials.

[32] Han F., Jiang J., Xu K., Wang N. (2019). Damage Detection of Common Timber Connections Using Piezoceramic Transducers and Active Sensing. Sensors.

[33] Hou Y., Hu W., Wang X., Hou T., Sun C. (2021). Damage Identification of Ancient Timber Structure Based on Autocorrelation Function. Adv. Civ. Eng..

[34] Petrović M., Pavićević D., Ilić I., Terzović J., Šekularac N. (2023). Elements of a Timber Lamella Structure: Analysis and Systematization of Joints. Buildings.

[35] Serdjuks D., Kurtenoks V., Tatarinovs A., Buka-Vaivade K., Lapkovskis V., Mironovs V., Podkoritovs A., Topcijs K. (2022). Non-Model Vibration Analysis Method for Health Monitoring of Structural Joints. Procedia Struct. Integr..

[36] Baas E.J., Riggio M., Barbosa A.R. (2021). A Methodological Approach for Structural Health Monitoring of Mass-Timber Buildings under Construction. Constr. Build. Mater..

[37] Zielińska M., Rucka M. (2021). Non-Destructive Testing of Wooden Elements. IOP Conf. Ser. Mater. Sci. Eng..

[38] Morgantini M., Betti R. (2020). The Inner Product Vector as an Output-Only Cross-Correlation-Based Feature to Structural Damage Assessment. J. Vibroeng..

[39] Saidin S.S., Jamadin A., Abdul Kudus S., Mohd Amin N., Anuar M.A. (2022). An Overview: The Application of Vibration-Based Techniques in Bridge Structural Health Monitoring. Int. J. Concr. Struct. Mater..

[40] Kurtenoks V., Kurajevs A., Buka-Vaivade K., Serdjuks D., Lapkovskis V., Mironovs V., Podkoritovs A., Vilnitis M. (2023). The Quality Assessment of Timber Structural Joints Using the Coaxial Correlation Method. Buildings.

